# Secondary Organising Pneumonia Among COVID-19 Patients: A Retrospective Case-Control Study

**DOI:** 10.7759/cureus.26230

**Published:** 2022-06-23

**Authors:** Joana Sinde, Tiago Teixeira, Cristóvão Figueiredo, Sofia Nunes, Daniel Coutinho, Inês Marques, Filipa Marques dos Santos, Sergio Campainha, Lurdes Santos, Luís Malheiro

**Affiliations:** 1 Medicine Department, Faculdade de Medicina da Universidade do Porto, Porto, PRT; 2 Infectious Diseases Department, Centro Hospitalar de Vila Nova de Gaia/Espinho, Vila Nova de Gaia, PRT; 3 Infectious Diseases Department, Centro Hospitalar de Vila Nova De Gaia/Espinho, Vila Nova De Gaia, PRT; 4 Radiology Department, Centro Hospitalar de Vila Nova de Gaia/Espinho, Vila Nova de Gaia, PRT; 5 Respiratory Medicine Department, Centro Hospitalar de Vila Nova de Gaia/Espinho, Vila Nova de Gaia, PRT

**Keywords:** corticosteroids, organising pneumonia, dexamethasone, sars-cov-2, covid-19

## Abstract

Coronavirus disease 2019 (COVID-19) is an infectious disease caused by the severe acute respiratory syndrome coronavirus 2 (SARS-CoV-2). Secondary organising pneumonia (OP) induced by SARS-CoV-2 infection is a recently recognised complication of COVID-19. We aimed to evaluate the prevalence of OP among hospitalised patients with COVID-19 pneumonia and to assess whether disease severity and other clinical factors and comorbidities are correlated with OP development.

We conducted a retrospective case-control study including hospitalised patients due to COVID-19 who performed a chest CT scan during hospitalisation and compared patients with clinical and radiological evidence of OP to patients without evidence of OP. Demographics, comorbidities, disease severity, dexamethasone/remdesivir treatment, laboratory results, and outcomes were compared between groups.

One hundred fifteen patients were included, of whom 48 (41.7%) fulfilled clinical and imaging criteria for OP. Among OP patients, the most common chest CT-scan findings were consolidations, arciform condensations, and subpleural bands. OP patients had longer hospitalisation (19.5 vs 10 days, p=0.002) and more frequent ICU admission, but no significant differences in readmittance or mortality rates within 180 days compared to controls. In the adjusted effects model, the need for supplementary oxygen on the 21^st^ day after symptom onset, the presence of Ordinal Scale for Clinical Improvement (OSCI) = 4, when compared to OSCI ≤ 3, and higher C-reactive protein on admission, were significantly associated with higher odds for OP. No other differences were identified between OP and controls after adjusting for other factors. The use of remdesivir or dexamethasone did not impact the diagnosis of OP. Only 38% of OP patients required treatment with high-dose corticosteroids.

In conclusion, SARS-CoV-2-induced OP may be more frequent than previously thought, especially among hospitalised patients and patients with a more severe disease, particularly those who fail to improve after the second week of disease or who present higher inflammatory markers on admission. It increases the length of stay, but not all patients require specific treatment and OP may improve despite the absence of high-dose corticosteroid treatment.

## Introduction

Coronavirus disease 2019 (COVID-19) is an infectious disease caused by the severe acute respiratory syndrome coronavirus 2 (SARS-CoV-2) [1]. It was first identified in the city of Wuhan in the Hubei province, China, in December 2019, where it is hypothesised to have emerged [2]. Since its appearance, several cases of SARS-CoV-2-induced organising pneumonia (OP) have been reported in patients presenting clinical deterioration following an initial period of symptomatic improvement [3].

OP is an anomalous process of pulmonary tissue repair with a characteristic histological pattern of lung damage with radiologic and clinical translation. It can be either cryptogenic (COP) when there is no identifiable cause or secondary when it is triggered by infections, medications, or pulmonary/systemic diseases [4,5]. The characteristic histological features of OP include intraluminal organising fibrosis in distal airspaces, with patchy distribution, preservation of lung architecture, uniform temporal appearance, and mild interstitial chronic inflammation [6]. This form of lung damage can be typically seen as bilateral or unilateral areas of consolidation with patchy distribution in chest radiography; however, high-resolution computed tomography (CT) is the gold standard imaging method for the diagnosis of OP. Numerous different radiological patterns can be observed and distribution is frequently diffuse or bilateral, although focal or unilateral abnormalities may be present. Most often, CT imaging reveals ill-defined patchy consolidation with a predominantly subpleural or peribronchial distribution [7]. OP is usually treated with corticosteroids [4].

The main objective of this study was to evaluate the prevalence of secondary OP among hospitalised patients with COVID-19 pneumonia and whether disease severity is correlated with OP development. We also questioned if dexamethasone treatment for COVID-19 pneumonia and/or patient’s clinical comorbidities influenced the risk of developing OP among hospitalised COVID-19 patients. Secondarily, we aimed to describe outcomes and radiologic findings of OP among COVID-19 patients.

## Materials and methods

Population and study design

We performed a retrospective case-control study with hospitalised patients due to COVID-19 at Centro Hospitalar de Vila Nova de Gaia/Espinho (CHVNG/E) during the period from January 1, 2021 to March 31, 2021. This study was approved by the CHVNG/E ethics committee (reference 125/2020-1) and a waiver of informed consent was obtained.

Inclusion criteria comprised age ≥ 18 years, laboratory confirmation of COVID-19 by positive nasopharyngeal nucleic acid amplification tests (NAAT) for SARS-CoV-2, need for hospitalisation, and performance of chest CT scan. Patients admitted for other conditions, surgical or medical, in which COVID-19 was not the main motive for hospitalisation were excluded. Patients with bacterial pneumonia or other concomitant lung infections were excluded. Readmitted patients were excluded to avoid case duplication.

OP was defined clinically and radiologically by the presence of suggestive chest CT-scan findings in combination with a compatible clinical presentation. Radiographic findings considered diagnostic of OP, following the American Thoracic Society/European Respiratory Society consensus for interstitial lung disease, included: classic consolidations (unilateral or bilateral patchy areas, either peripheral, subpleural, or peribronchovascular) and nodules. In addition, other findings consistent with OP diagnosis were the reversed halo (attol) sign, perilobular abnormalities (arciform condensations), bands of consolidation, crazy paving, and progressive fibrotic pattern [5,8]. The presence of ground-glass opacities, in the absence of other findings, was considered diagnostic of COVID-19 pneumonia but not OP.

Lung infections were excluded by proper clinical and laboratory evaluation which included a combination of low inflammatory biomarkers, with procalcitonin < 0.5 mcg/L and C-reactive protein < 10 mg/dL, and a negative septic screening which included urinary pneumococcal and Legionella antigens, oropharyngeal NAAT for respiratory syncytial virus and influenza, sputum and blood cultures, in the absence of antibacterial treatment.

Data collection

Participants were identified using the CHVNG/E COVID-19 registry database. Data were retrospectively retrieved from electronic medical records and included demographics (sex and age), smoking habits, medical comorbidities (chronic obstructive pulmonary disease, diabetes mellitus, arterial hypertension, dyslipidaemia, cardiopathy, atrial fibrillation, obesity, or autoimmune disease), laboratory parameters at admission including: leucocyte count, neutrophil and lymphocyte count, C-reactive protein (CRP), interleukin-6 (IL-6), ferritin, procalcitonin, lactate dehydrogenase (LDH), troponin T, serum creatinine, creatinine kinase, aspartate transaminase (AST), alanine transaminase (ALT), fibrinogen and D-dimer, clinical frailty score [9], need for oxygen supplementation and respective duration, duration of symptoms when chest CT-scan was performed, need for intensive unit (ICU) admission, treatment with remdesivir/dexamethasone for COVID-19, or the need for corticosteroids for OP (0.75-1 mg/kg prednisolone per day during three months as recommended by BTS 2008 guidelines [10]), worst World Health Organisation Ordinal Scale for Clinical Improvement (OSCI) score while hospitalised [11], length of hospitalisation, readmittance within 180 days and death within 180 days.

Statistical analysis

Data processing and statistical analysis were performed using IBM SPSS Statistics®, software version 27 (IBM Corp., Armonk, NY). The significance level for all tests was defined as p<0.05. Categorical data were presented as proportions (%), continuous data with normal distribution were presented as means ± standard deviation, and continuous data with non-normal distribution were presented as medians and quartiles (Q_1_; Q_3_).

For the main objective of this study, two groups were compared: patients with evidence of OP and patients without evidence of OP. For the univariate analysis, the χ^2^ test was used for dichotomic categorical variables, the one-way ANOVA test was used for categorical variables with more than two categories, the t-test was used for normally distributed continuous variables, and the Mann-Whitney U test was used for non-normally distributed continuous variables. A multivariate analysis was then performed using logistic regression in which only variables with a p-value < 0.10 on univariate analysis were included. Odds ratio (OR) with a 95% confidence interval (CI) were used to describe the results. Only variables with more than 90% available results were considered in the multivariate model due to limitations in sample size. In order to minimise missing data bias, all medical record entries were reviewed to avoid missing data due to incomplete records, and all chest CT scans were revised by a second radiologist to ensure there were no missing OP diagnoses.

## Results

**Figure 1:**
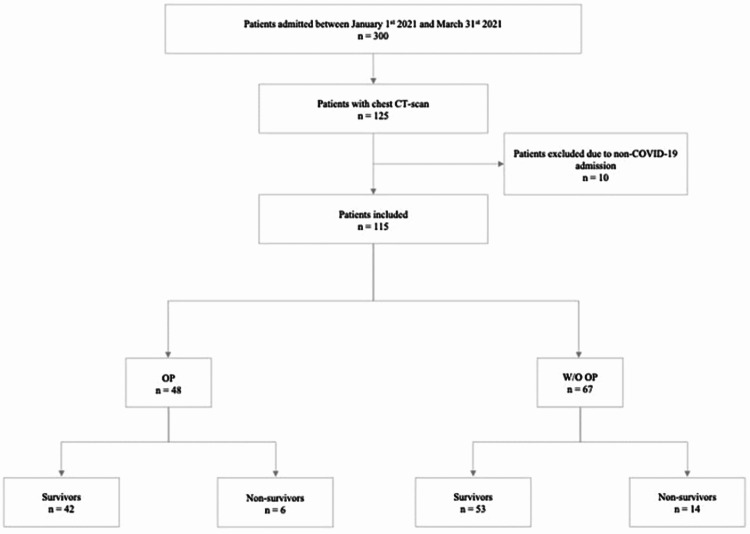
Patient allocation flow diagram OP - organising pneumonia, W/O - without

**Figure 2:**
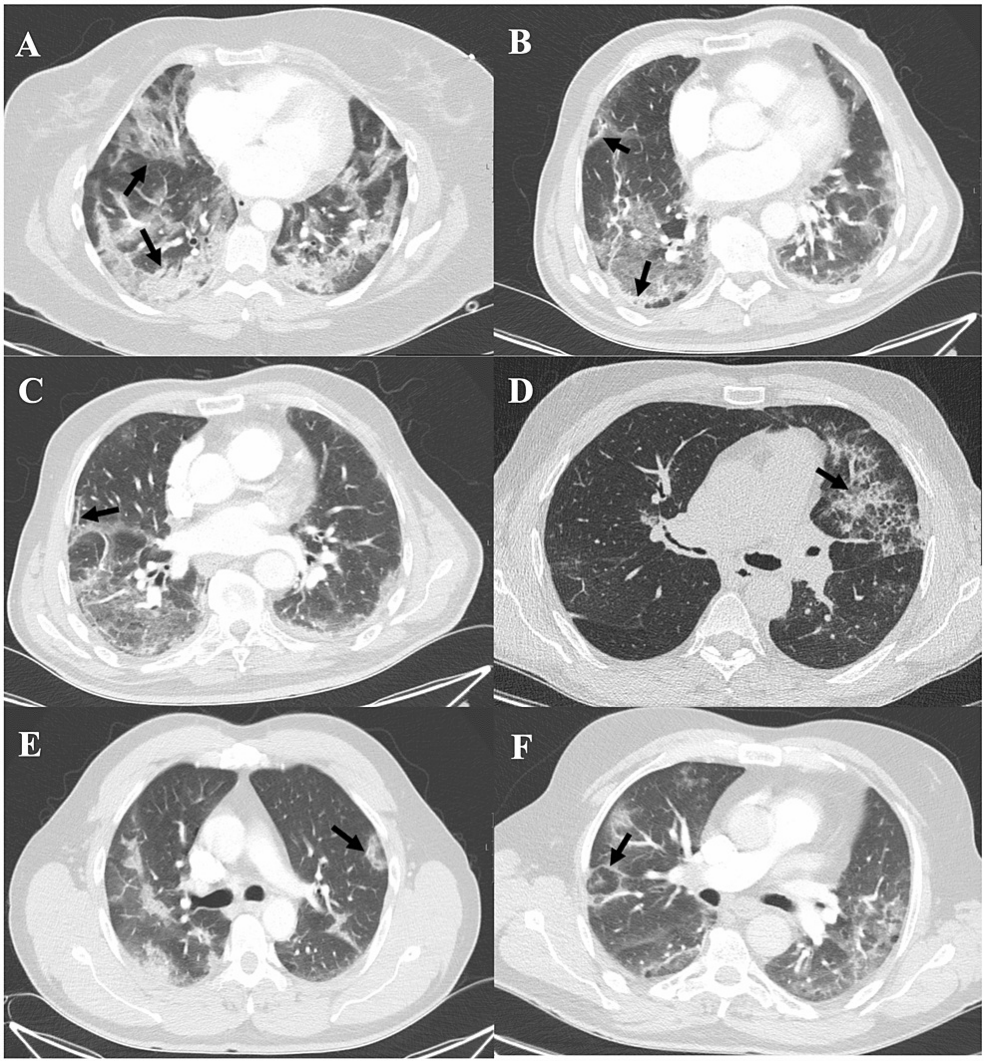
CT features of organising pneumonia in COVID-19 pneumonia patients (A) Bilateral subpleural and peribronchovascular consolidations (black arrow). (B) Subpleural linear consolidation (black arrow). (C) Parenchymal bands (black arrow). (D) Crazy paving pattern (ground-glass opacities with inter and intralobular septal thickening) (black arrow). (E) Perilobular opacities (ill-defined perilobular linear opacities, thicker than the thickened interlobular septa with an arch shape) (black arrow). (F) Reversed halo (attol) sign (central ground-glass opacity surrounded by denser consolidation of crescentic shape) (black arrow).

During the study period, 300 patients were admitted with the diagnosis of COVID-19. Of these, 125 required chest CT imaging during their hospital stay (Figure 1). 10 (8%) patients were excluded as the reason for admission was other than COVID-19. Of the 115 included patients, 48 (41.7%) fulfilled clinical and imaging criteria for OP. Within the 48 patients diagnosed with OP, the most common chest CT-scan findings were consolidations, arciform condensations, fibrotic features, and subpleural bands (Table 1, Figures 2A-2F). Most patients with OP presented either one (35.4%), two (33.3%), or three (22.9%) distinctive radiographic findings, with a minority presenting four (6.2%) or five (2.0%) different radiographic findings.

**Table 1:** Radiographic findings of OP among COVID-19 patients OP - organising pneumonia

	Total n = 48	Time from symptoms to CT-scan (days)		P-value
		< 14 (n = 15)	≥ 14 (n = 33)	
Suggestive consolidations	41 (85.4%)	12 (80%)	29 (87.9%)	0.662
Arciform condensations	30 (62.5%)	10 (66.7%)	20 (60.6%)	0.757
Fibrotic features	11 (22.9%)	1 (6.7%)	10 (30.3%)	0.073
Subpleural bands	10 (20.8%)	3 (20.1%)	7 (21.2%)	1.000
Reversed halo sign	3 (6.3%)	2 (13.3%)	1 (3.0%)	0.227
Crazy paving	1 (2.1%)	0	1 (3%)	1.000

The mean age of the study population was 67.9 years and the patients were predominantly male (63.5%). The three most commonly identified medical comorbidities were arterial hypertension (57.4%), dyslipidaemia (48.7%), and diabetes mellitus (32.2%). Approximately one in four patients had a pre-admission frailty score of four or higher. During the hospital stay, 100 patients (87%) required supplementary oxygen, and the median length of oxygen supplementation was 11 days (6; 18.5); of these, 40 (34.8%) required high flow oxygen therapy. 29 patients (25.2%) were admitted to the Intensive Care Unit. During hospitalisation, 13 patients (11.3%) and 95 patients (82.6%), received treatment with remdesivir (200 mg intravenous loading dose, followed by 10 mg per day for 5-10 days) and dexamethasone (6 mg intravenous per day), respectively. Approximately half of the patients (47.8%) performed a chest CT scan within the first 14 days after symptoms onset. Intra-hospital mortality was 9.6% (11 patients) and 30-day mortality was 15.7% (18 patients) (Table 2).

**Table 2:** Univariate analysis of risk factors for developing organising pneumonia among hospitalised COVID-19 patients AHT - arterial hypertension, ALT - alanine transaminase, AST - aspartate transaminase, COPD - chronic obstructive pulmonary disease, CRP - C-reactive protein, DM - diabetes mellitus, ICU - intensive care unit, IL-6 - Interleukin-6, LDH - lactate dehydrogenase, N-to-L Ratio - neutrophil to lymphocyte ratio, O_2_ - oxygen, OP - organising pneumonia, OSCI - Ordinal Scale for Clinical Improvement, Sup. - supplementary, Sx. - Symptoms, TLC - total leukocyte count

		Total n = 115	Organising Pneumonia		P-value
			Yes (n = 48)	No (n = 67)	
Sex	Female	42 (36.5%)	13 (27%)	29 (43%)	0.075
	Male	73 (63.5%)	35 (73%)	38 (57%)	
Age (years)		67.86 (12.96)	68.0 (11.2)	67.7 (14.2)	0.888
Frailty Score ≥4		29 (25.2%)	6 (12.1%)	23 (34.3%)	0.008
COPD		16 (13.9%)	6 (13%)	10 (15%)	0.711
DM		37 (32.2%)	17 (35%)	20 (30%)	0.529
AHT		66 (57.4%)	27 (56%)	39 (58%)	0.834
Dyslipidaemia		56 (48.7%)	26 (54%)	30 (45%)	0.320
Any cardiopathy		26 (22.6%)	6 (13%)	20 (30%)	0.028
Atrial fibrillation		12 (10.4%)	1 (2%)	11 (16%)	0.013
Obesity		30 (26.1%)	13 (27%)	17 (25%)	0.837
Autoimmune disease		5 (4.3%)	2 (4%)	3 (4%)	1.000
Smoking habits		24 (20.9%)	9 (19%)	15 (22%)	0.636
Supplementary O_2_		100 (87.0%)	46 (95.8%)	53 (79%)	<0.001
Duration of Sup. O_2_ (days)		11 (6; 18.5)	16 (10; 25)	7 (5; 14)	<0.001
Sup. O_2_ (7th day of Sx.)		47 (40.9%)	23 (48%)	24 (36%)	0.193
Sup. O_2_ (14th day of Sx.)		69 (60.0%)	38 (79%)	31 (46%)	<0.001
Sup. O_2_ (21st day of Sx.)		43 (37.4%)	29 (60%)	14 (21%)	<0.001
Worst OSCI	≤3	16 (13.9%)	2 (4.2%)	14 (20.9%)	Ref.
	4	61 (53.0%)	29 (60.4%)	32 (47.8%)	0.032
	≥5	38 (33.0%)	17 (35.4%)	21 (31.3%)	0.065*
Day of Symptoms when CT-scan	<14	55 (47.8%)	15 (31.2%)	40 (59.7%)	0.003
	≥14	60 (52.2%)	33 (68.8%)	27 (40.3%)	
ICU admission		29 (25.2%)	17 (35%)	12 (18%)	0.033
Remdesivir		13 (11.3%)	7 (15%)	6 (9%)	0.347
Dexamethasone		95 (82.6%)	46 (96%)	49 (73%)	0.002
TLC (per µL) (n=115)		6420 (5010; 6420)	6635 (5302; 10267)	6060 (4580; 9010)	0.234
N-to-L Ratio		5.74 (3.32; 8.51)	6.01 (4.65; 9.46)	5.67 (2.84; 8.01)	0.567
Platelets (x10^3^/µL) (n=115)		229 (94)	230 (82.9)	229 (102)	0.932
CRP (mg/dL) (n=115)		8.62 (3.57; 8.62)	10.9 (6.60; 19.0)	5.46 (2.6; 8.6)	<0.001
IL-6 (pg/dL) (n=52)		22.1 (11.1; 68.8)	22.3 (11.1; 103.0)	19.0 (10.6; 56.8)	0.402
Ferritin (mcg/L) (n=114)		1011 (526; 1540)	1404 (685; 1929)	705 (400.5; 1282)	<0.001
Procalcitonin (mcg/L) (n=106)		0.12 (0.05; 0.36)	0.19 (0.07; 0.67)	0.09 (0.05;0.22)	0.019
LDH (U/L) (n=114)		319 (246; 395)	359 (285; 442)	292 (207; 363)	0.002
Troponin T (ng/mL) (n=95)		14 (8; 28)	10 (8; 25.5)	16.5 (9; 31.5)	0.231
Serum creatinine (mg/dL) (n=115)		0.80 (0.65; 1.14)	0.81 (0.68; 1.13)	0.80 (0.61; 1.16)	0.642
Creatinine Kinase (U/L) (n= 97)		91 (53; 136)	98 (55.8; 133.5)	80 (51.0; 145.0)	0.980
AST (U/L) (n=114)		28 (18; 49)	38 (28.5; 52.75)	29.5 (21.0; 37.8)	0.003
ALT (U/L) (n=113)		31 (23; 47)	31 (21.0; 53.8)	27 (15.0; 47.0)	0.178
Fibrinogen (mg/dL) (n=108)		586 (150)	570 (157)	589 (141)	0.057
D-Dimer (mcg/mL) (n=112)		1.31 (0.81; 2.53)	1.56 (0.84; 2.53)	1.19 (0.76; 2.57)	0.221
Length of stay (days)		14.00 (7; 14)	19.5 (11; 31)	10 (6; 18)	0.002
Readmittance in 180 days		13 (11.3%)	4 (8%)	9 (13%)	0.394
Death by day 30		18 (15.7%)	5 (10%)	13 (19%)	0.191
Death by day 180		20 (17.4%)	6 (13%)	14 (21%)	0.241
Treatment for OP		18 (15.7%)	18 (38%)	0	NA
*p = 0.965 when OSCI ≥ 5 compared to OSCI 4					

The group of patients who developed OP had a higher proportion of male patients (73% vs 57%), but this difference was not statistically significant. The mean age was approximately the same in both groups. The proportion of vulnerable patients (Frailty Score ≥4) among those who developed OP was significantly lower than the control group (12.1% vs 34.3%). Among the evaluated comorbidities, there were no significant differences between groups, except for cardiopathy and atrial fibrillation which were significantly less frequent among patients who developed OP (13% vs 30% and 2% vs 16%, respectively). The proportion of patients requiring supplementary oxygen was significantly higher in the study group (98% vs 79%); only two patients in this group did not require oxygen therapy. In addition, the duration of oxygen therapy was significantly longer (16 vs 7 days) in the OP group. The proportion of patients requiring oxygen supplementation during the first week after symptom onset was not significantly different between groups; however, by the end of the second and third weeks, oxygen requirement was significantly more frequent among the OP group (79% vs 46% and 60% vs 21%, respectively). There was also a statistically significant difference regarding clinical severity: patients with OSCI = 4 were more likely to develop OP than patients with OSCI ≤ 3; however, there was no statistically significant difference in the development of OP among patients with OSCI ≥ 5 when compared with patients with OSCI ≤ 3 (p=0.065), or patients with OSCI = 4 (p=0.965). On admission, the OP group had significantly higher CRP, ferritin, procalcitonin, LDH, and AST in comparison to the control group.

A statistically significant difference regarding the timing of chest CT-scan between groups was found, as most (59.7%) of the patients diagnosed with OP had a chest CT scan performed more than 14 days after symptom onset, while most (68.8%) of the patients in the control group had it performed in the first 14 days after symptom onset.

Regarding treatment for COVID-19, there was no difference in the frequency of remdesivir administration. Dexamethasone therapy, however, was significantly less common among patients in the control group (96% vs 73%). The hospitalisation was significantly longer among OP patients (19.5 vs 10 days) and these patients more frequently required ICU admission, but there was no statistically significant difference in readmittance or mortality rates within 180 days between groups.

In the adjusted effects model, the need for supplementary oxygen on the 21^st^ day after symptom onset, the presence of OSCI = 4, when compared to OSCI ≤ 3, and higher CRP, were significantly associated with higher odds for OP (Table 3). We excluded procalcitonin and fibrinogen from this analysis as they were not systematically collected, and inclusion would decrease the sample size in this analysis. Our model predicts that, after adjusting for other variables, if the patient still needs supplementary oxygen on the 21^st^ day after symptom onset, the odds of being diagnosed with OP are 7.033 times higher. Our findings were still significant, even after adjusting for the timing of chest CT-scan performance. Additionally, if the worst OSCI = 4, the odds for OP are 2.722 times higher than having OSCI ≤ 3. After adjusting for other factors, no differences were found between groups for sex, frailty score, comorbidities, and the use of dexamethasone as a treatment for COVID-19.

**Table 3:** Multivariate analysis of risk factors for developing organising pneumonia among hospitalised COVID-19 patients AST - aspartate transaminase, CI - confidence interval, CRP - C-reactive protein, LDH - lactate dehydrogenase, O_2_ - oxygen, OR - odds ratio, OSCI - Ordinal Scale for Clinical Improvement, Sup. - supplementary, Sx. - symptoms

	OR	P-value	95% CI for the OR
Sex - male	0.560	0.359	0.162 - 1.933
Frailty Score ≥4	0.521	0.330	0.140 – 1.933
Any cardiopathy	0.205	0.074	0.036 – 1.166
Auricular fibrillation	0.403	0.556	0.020 – 8.300
Worst OSCI ≤3	Ref.	0.014	Ref.
Worst OSCI = 4	2.722	0.047	1.014 – 7.313
Worst OSCI ≥5	0.363	0.086	0.114 – 1.154
Sup. O_2_ (14^th^ day of Sx.)	3.312	0.058	0.952 - 11.433
Sup. O_2_ (21^st^ day of Sx.)	7.033	0.009	1.640 – 30.153
Day of Symptoms when CT-scan <14 days	1.249	0.713	0.383 – 4.077
Dexamethasone	1.010	0.993	0.112 – 9.124
CRP (mg/dL)	1.103	0.028	1.011 – 1.203
Ferritin (mcg/L)	1.001	0.122	1.000 – 1.001
LDH (U/L)	1.001	0.730	0.995 – 1.007
AST (U/L)	1.000	0.964	0.980 – 1.019

## Discussion

Our results show a high prevalence (41.7%) of OP among hospitalised patients with COVID-19 pneumonia. The true prevalence of this condition remains unclear, as it varies in accordance with population setting and very limited data has been published about OP due to COVID-19. For instance, Myall et al. found radiological evidence of interstitial lung disease, predominantly OP, in 4.8% of 837 patients hospitalised due to SARS-CoV-2 pneumonia, six weeks after discharge. In that study, 83% of the patients diagnosed with OP previously required supplementary oxygen during the pneumonia phase of COVID-19, and half of them required ICU admission with 46% requiring invasive mechanical ventilation. Contrarily, among critically ill patients with COVID-19 pneumonia, admitted to an ICU, Rocha et al.described a prevalence of OP that reached 58.0% [12,13]. Both studies suggested that a more severe disease may be associated with progression to OP.

In our setting, a high proportion of patients (185 out of 300) did not perform CT scans during their hospital stay. Due to limited resources, patients with more severe disease presenting with respiratory failure were more likely to be offered a chest CT scan than those with mild disease, in whom imaging tests would not justify a change in approach as long as there was a clinical improvement. Although this criterion complies with the American College of Radiology's recommendations for the use of CT-scan in COVID-19 patients [14], it may have led to an overestimation of the overall prevalence of OP, as patients with mild disease were underrepresented in our study population. Nonetheless, we found significantly higher odds for OP in patients requiring supplementary oxygen and in patients admitted to the ICU when compared to those with mild disease (OSCI ≤ 3), which is consistent with the results found by the studies presented above.

However, patients requiring high-flow nasal cannula or non-invasive/invasive ventilation (OSCI ≥ 5) did not have significantly higher odds for OP when compared to patients with low-flow oxygen. This may be partially explained by a selection bias, as mortality among patients with critical COVID-19 is expected to be higher and, therefore, some patients may have died before performing a chest CT scan and being diagnosed with OP. During the study period, local ICU occupation rates were at the highest level recorded since the beginning of the pandemic, primary vaccination coverage for SARS-CoV-2 only included healthcare workers and patients ages ≥ 80 years, which are underrepresented in the study population, and ICU mortality within our hospital was estimated to be around 14%. Another point supporting the assumption that higher clinical severity may increase the odds for OP is the significant association between higher CRP and higher odds for OP. As CRP is a well-known marker of COVID-19 severity, its independent association with OP development suggests that higher systemic inflammation on admission may be predictive of developing OP. This may also explain why patients with higher frailty scores presented a lower prevalence of OP, suggesting that inability to develop a robust inflammatory response against COVID-19 may decrease the progression to OP.

By the time of study conduction, dexamethasone 6 mg/day was the standard-of-care for patients with COVID-19 requiring supplementary oxygen, following the published results of the RECOVERY trial [15]. Remdesivir was also part of the prescribed regimen for a selected population in need of supplementary oxygen if started during the first week after symptom onset. Remdesivir decreases viral SARS-CoV-2 replication while dexamethasone is believed to modulate immune response and prevent COVID-19 progression by decreasing the inflammatory response characteristic of severe lung injury. Corticosteroids are also the main treatment for OP [10], which raises the question of whether patients who received dexamethasone would have a lower risk of developing OP. However, in our findings, neither treatment resulted in decreased risk of OP.

Awareness seems to be the most important aspect in diagnosing OP. Literature reports that up to 70% of patients with OP will respond to adequate corticosteroid treatment and OP represents an independent predictor of good prognosis, hence high index of suspicion must be kept to assure a timely diagnosis and adequate treatment implementation [16]. Timely treatment might also help to prevent persistent functional deficits [12]. Although other authors described OP later in the disease course, well after COVID-19 resolution [12], our results show that at least 30% of the OP cases where evident in chest CT-scan performed in the first 14 days after symptom onset, suggesting an early progression from ground glass opacities to a consolidative or arciform pattern [4]. Some patients who performed CT-scan later in the disease course presented fibrosis and traction bronchiectasis, suggesting that the untreated interstitial disease may have progressed to a fibrotic phase, although it is not possible to exclude the presence of chronic interstitial lung disease as patients did not have a chest CT-scan prior to the hospital admission. This means the likelihood of missing diagnosis OP cases is low, as literature reports that maximum SARS-CoV-2 induced lung injury is achieved around 10 to 11 days after symptom onset and resolution tends to be gradual over up to four weeks [12,17].

Our population’s average age is in accordance with literature evidence that patients with OP (similarly cryptogenic and secondary) typically present between fifth and sixth decade of life, however it did not differ from patients without OP. Contrarily, there was a predominant proportion of male patients in our OP population, while literature suggests an equal sex distribution [4]. However, there was no statistically significant difference in the proportion of male patients between case and control groups. This may represent a bias by the use of a hospitalised population sampling method, as male sex is a known risk factor for the development of severe COVID-19 [18].

We did not find a statistically significant difference regarding smoking habits between groups. Literature is contradictory regarding this topic. The American Thoracic Society/European Respiratory Society International Multidisciplinary Consensus Classification of the Idiopathic Interstitial Pneumonias reports a 2:1 ratio of non-smokers to smokers among patients with COP; while Drakopanagiotakis et al. observed a prevalence of smoking habits of 56% among patients with OP and no difference between COP and secondary OP groups [4,6].

Although the presence of OP was not associated with higher mortality or readmittance rates, we observed a statistically significant longer hospitalisation among OP patients, mostly due to longer needs of supplementary oxygen. Our findings are consistent with literature reports of OP representing a benign condition and an independent predictor of good prognosis when treatment is started early [16]. In our study, the need for supplementary oxygen on the 21^st ^day after symptom onset was the strongest predictor for the presence of OP. This finding suggests that, in clinical practice, the clinician should look for OP with a chest CT-scan when the patient fails to improve after the second week of symptoms, in the absence of other causes, as a specific treatment may be warranted if OP is diagnosed.

There are some limitations in our study. The single-centre nature and relatively small sample size limit the generalisability of our results. As a retrospective study, there were variables with frequently missing data, including relevant data such as comorbidities and smoking habits. Variables of interest, such as procalcitonin, fibrinogen and IL-6, which literature suggests to be associated to COVID-19 disease severity, were not systematically collected and were, therefore, excluded from the multivariate analysis [13,18].

Additionally, as our study population was limited to hospitalised patients, a potential selection bias of severe cases of both COVID-19 and OP may have occurred. Patients with less severe symptoms are less likely to seek professional help and be hospitalised, consequently there may have been an underdiagnosis of less severe OP among patients with less severe COVID-19.

The clinic and radiographic presumptive diagnosis in the absence of histological confirmation of OP is another limitation of this study. Many findings in OP, especially consolidations, are also characteristic of other conditions such as bacterial pneumonia. However, literature suggests that high-resolution CT presenting adequate signs of disease allows diagnosis in up to 80% of cases [19]. Therefore, performing bronchoscopy with transbronchial biopsies or surgical lung biopsies in critically ill patients, who can be empirically treated after clinic and radiographic presumptive diagnosis, may be ethically questionable. On the other hand, the pandemic context in which the study was conducted, greatly limited timely access to bronchoscopy, reinforcing the need for presumptive diagnosis.

Furthermore, regarding optimal treatment for SARS-CoV-2 induced OP, large scale studies are still necessary to determine optimal timing, dosing, and duration of corticosteroid treatment. Of the 48 patients diagnosed with OP, only 18 (38%) required specific treatment with high-dose corticosteroids, while most patients improved with standard dose dexamethasone. As such, it is questionable if the diagnosis of less severe cases of OP is needed, as many will improve without intervention, routine chest CT-scan will increase costs and there is no scientific evidence that increasing corticosteroid dose will have a positive impact for these patients. Further investigations, with follow up after hospital discharge and evaluation of functional impact of OP among COVID-19 patients are required to better understand disease progression and possible long-term effects.

## Conclusions

Secondary OP is a complication of COVID-19 that may be more frequent than previously thought, especially when hospitalised patients with SARS-CoV-2 pneumonia are considered. OP may also be more common in patients with more severe diseases, particularly those who fail to improve from respiratory insufficiency after the second week of disease or who present higher inflammatory markers on admission. It increases the length of stay, but not all patients require specific treatment and may even improve despite the absence of high-dose corticosteroid treatment. Further studies should aim to determine the optimal treatment for SARS-CoV-2 induced secondary OP and evaluate the functional impact of OP among COVID-19 patients in both the short and the long terms.
